# Multigravida Women With Moderate to Severe Anaemia in Third Trimester: Fetomaternal Outcomes

**DOI:** 10.7759/cureus.20493

**Published:** 2021-12-17

**Authors:** Kruti Savaliya, Nalini Sharma, Rushikesh Surani, Vimla Dhakar, Arun Gupta

**Affiliations:** 1 Obstetrics and Gynaecology, Pandit Deendayal Upadhyay Medical College and Civil Hospital, Rajkot, IND; 2 Obstetrics and Gynaecology, Geetanjali Medical College and Hospital, Udaipur, IND

**Keywords:** perinatal mortality, maternal death, hypertension pregnancy induced, iron, infant low birth weight, pregnancy outcome, pregnancy trimester third, parity, gravidity, anemia

## Abstract

Background & Objectives: Anaemia has been reported to be associated with adverse pregnancy outcomes, especially when presenting in the last trimester. In addition to prevalent common causes of anaemia in pregnant women, poor replenishment of iron stores after a pregnancy event is specific to women with higher birth orders. Anaemic women presenting in the third trimester are more prone to maternal complications such as infections, toxaemia, antepartum haemorrhage, cardiac failure, pre-eclampsia as well as fetal hazards too such as low birth weight, pre-term deliveries, developmental anomalies, and even neonatal death. When presented near term there are higher chances of feto-maternal morbidity and mortality. In the current study, analysis is done of feto-maternal outcomes, routes of delivery of women, causes of anaemia in multigravida women in the third trimester suffering from moderate to severe anaemia in a tertiary care centre of Western Rajasthan of India.

Methods: A prospective observational clinical study was conducted on patients attending Geetanjali Hospital over a period of 18 months. A total of 70 consecutive multigravida pregnant women having moderate to severe anaemia in the third trimester were selected. Statistical analysis of the data collected was done and a p-value <0.05 was taken as significant.

Results: Moderate and severe anaemia in the study population were 44.28% and 55.71%, respectively. The mean haemoglobin level of all study groups was 7.0 gm%. Pre-eclampsia, placenta praevia, postpartum haemorrhage (PPH), congestive cardiac failure (CHF), neonatal intensive care unit (NICU) admission, preterm birth (PTB), low birth weight (LBW), intrauterine death (IUD), low Appearance, Pulse, Grimace, Activity, and Respiration (APGAR) score, and birth asphyxia records were investigated. Of the patients studied, 18.57% had PPH, 15.71% had pre-eclampsia, 8.57% had IUD, and 37.14% newborns were LBW.

Interpretations and Conclusion: Multiparity itself is a major risk factor of anemia. Anemia presenting in the third trimester of pregnancy is a proxy indicator of care received by gravid women in the early antenatal period. In combination, a multigravida in the third trimester with less time to restock iron and vitamin stores may result in considerable maternal as well as perinatal mortality and morbidity.

## Introduction

Anaemia is defined by the WHO as haemoglobin level ≤ 11 g/dl in pregnant women [1]. Anaemia is divided into three categories according to haemoglobin level: mild (9.1 to 11gm%), moderate (7.1 to 9 gm%), severe (≤ 7 gm%). Of the maternal deaths due to anaemia in South Asia, 80% occur in India [1]. 

Factors contributing to anaemia in obstetrics are: increased physiological and fetal demands, inadequate intake, poor absorption due to endemic diseases, viz., malaria, hookworm infestation, and blood loss because of multiple pregnancy losses or during and after labour [2]. All iron demands of the growing foetus are fulfilled either from maternal iron stores or absorption of iron from the maternal diet. Each obstetric event creates a considerable deficit in iron stores, which, if not actively replenished, precipitates anaemia in closely spaced pregnancies, i.e in multigravida women. This is the basic contrast between higher birth order pregnancies and primiparous females.

Anaemia in pregnancy gives rise to a plethora of maternal and fetal health issues. It can lead to multiple abortions, pre-eclampsia, recurrent infections, antepartum haemorrhage, cardiac failure in the antenatal period. Post-partum haemorrhage (PPH), wound gaping, delayed involution, and puerperal sepsis diminish the joy of the postpartum period. Few among the multitude of hazards faced by the foetus are intra-uterine growth retardation, intrauterine death (IUD), preterm birth (PTB), low birth weight (LBW), neonatal morbidity (developmental delays, recurrent infections, repeated hospital visits), and even neonatal mortality. There is a paucity of studies focussing on third-trimester anaemia in multigravida women. The current study analyses causes, risk factors, frequency, and feto-maternal outcomes of moderate to severe anaemia (according to WHO guidelines) in third-trimester multigravida pregnancy [1].

## Materials and methods

This was a hospital-based prospective and observational analytical study conducted in the department of Obstetrics and Gynaecology at Geetanjali Medical College and Hospital, Udaipur. Out of 93 women enrolled for this probe, 23 women were lost to follow-up. A total of 70 multigravida women reporting to the antenatal clinic in the third trimester having clinical/laboratory signs of moderate to severe anaemia were taken as study subjects. The study duration was 18 months from February 2019 to July 2020. Human Research and Ethics Committee, Udaipur, Rajasthan, issued approval GU/HREC/EC/2019/606.

The study aimed to study the frequency, causes, risk factors, maternal and fetal outcome of moderate to severe anaemia in third-trimester multigravida pregnancy. Out of the total patients during the study duration, the current study included all the women who fulfilled the inclusion criterion: multigravida females (gravida 3 and more) of 28 to 40 weeks of gestation having clinical features suggestive of moderate to severe anaemia with Hb 7.1 - 9 gm% (moderate) and ≤ 7 gm% (severe) anaemia, giving informed consent to participate in the study. Exclusion criteria were antenatal females of <28 weeks of gestation not willing to follow up. The women were interviewed in their own language in full detail regarding age, literacy, socioeconomic status (as per Kuppuswamy socioeconomic scale), diet, etc. Proforma was prepared in English, which was clinician-administered and translated to local language while delivering. It was validated internally. Pregnancy details regarding significant past and family history were noted. A thorough general physical, systematic, and obstetrical examination was conducted for each subject. 

The approach to anaemia included identification of the type of anaemia. History of poor dietary intake, strictly vegetarian diet, smoking, alcoholism, pagophagia, bleeding haemorrhoids, gastric bypass surgery, any chronic illness such as tuberculosis/malaria/diabetes/chronic kidney disease, intake of drugs such as deworming agents, phenytoin, phenobarbitone, and trimethoprim-sulphamethoxazole, and complaints of easy fatiguability or weakness was elicited. Previous pregnancy ailments were interrogated such as anaemia, puerperal sepsis, pre-eclampsia, and blood transfusion. Pallor, glossitis, splenomegaly, jaundice were noticed on general physical examination. RBC indices - haemoglobin/hematocrit level, mean corpuscular volume (MCV), mean corpuscular haemoglobin (MCH), mean corpuscular haemoglobin concentration (MCHC) simultaneous with peripheral blood film were analysed. In peripheral blood film (PBF), microcytic hypochromasia, anisopoikilocytes, megaloblasts, target cells, Howell-Jolly bodies, sickle-shaped cells, and malarial parasite were searched for. Raised serum transferrin level pointed towards iron deficiency anaemia and low serum Iron levels taken were <60 microgram/mL. When iron levels were found high, haemoglobin electrophoresis was done together with PBF to draw a distinction between thalassemia and sickle cell disease. Serum ferritin levels <30 micrograms/L taken as the earliest marker of depleted iron stores. Also, it aided to differentiate between anaemia of chronic disease from iron deficiency anaemia. Plasma bilirubin and plasma haptoglobin were sent to identify haemolytic anaemia. Urine microscopy was done. Stool examination for occult blood was conducted for hookworm infestations. Leukocyte count, RBC count, platelet count and reticulocyte count helped to discern leukaemias. The fetal wellbeing was ensured by obstetrical examination as well as ultrasonogram on admission.

All the study subjects were carefully followed in the antepartum, intrapartum, and postpartum periods (till they received their discharge from the hospital). Dietary sources of iron were discussed in detail and assertions were made for regular use of hematinics. Enquiries were made regarding new symptoms, which were confirmed by clinical examination or laboratory investigations to detect the obstetrical and neonatal difficulties due to anaemia at the earliest. Women in labour were carefully monitored and progress of labour was noted. They were also watched for any obstetric or medical complexity, which was dealt with efficiently. Blood transfusion was the commonest mode of treatment resorted to in severe anaemia cases and was done in antepartum, intrapartum, or postpartum periods. Utmost care was exercised to prevent the complications of blood transfusion. Blood components were used in all the cases. Finally, the mode of delivery, operative intervention, maternal morbidity and mortality were recorded in all the study subjects.

Breastfeeding was encouraged immediate post-partum. Emphasis was laid on the maintenance of personal hygiene and early ambulation to avoid complications in the puerperium. The perinatal outcome (breastfeeding, live birth, stillbirth, IUD, NICU admission) were taken note of. The weight of newborns and their Appearance, Pulse, Grimace, Activity, and Respiration (APGAR) scores at one and five min were documented. The neonate was attended by the paediatrician to detect the complications of severe anaemia. The outcome was judged by analysis of the above data.

Need for sterilization was addressed during antenatal visits in all females. Willing participants were guided accordingly. Advice was given regarding the need for continuous use of haematinics for a minimum period of six months post-partum. Importance of the same was explained to the patient as well as available family members.

Data entry was done in Microsoft Excel Windows 10 (Microsoft Corp., Redmond, Washington). Data Analysis was done with IBM SPSS Statistics for Windows, Version 21.0 (Released 2012; IBM Corp., Armonk, New York). Analysis of data was done with percentage, proportion, mean, and using Chi-square test and Fischer exact test. p-value <.05 is taken as significant.

## Results

As shown in Table 1, the majority of the study subjects (37.14%) belonged to the age group of 30-34 years. The minimum age in the entire study group was 23 years and the maximum age was 48 years. Mean age of all study subjects was 33 years. Of the women, 64.28% belonged to lower socio-economic status, 22.85% belonged to middle socio-economic status group, and 12.85% belonged to upper class. Thus, poor nutrition was the leading cause of anemia. It was observed that 67.14% women were illiterate. While 52.86% were from rural areas, 47.14% were from urban areas, showing that urban mothers were also equally anaemic. Of the study subjects, 30% were not booked cases; 70% were booked cases visiting antenatal clinic of our institution.

**Table 1:** Socio-demographic factors affecting anaemia

Socio demographic Factor		
Age	Number(n=70)	%
20-24 years	6	8.57%
25-29 years	16	22.86%
30-34 years	26	37.14%
35-39 years	10	14.28%
40-44 years	8	11.42%
45-49 years	3	4.29%
Socioeconomic Class	Number (n=70)	%
Lower Class	45	64.28%
Middle Class	16	22.85%
Upper Class	9	12.85%
Educational Status	Number (n=70)	%
Illiterate	47	67.14%
Literate	23	32.85%
Residence	Number (n=70)	%
Rural	37	52.86%
Urban	33	47.14%
Diet Pattern	Number (n=70)	%
Mixed diet	20	28.57%
Vegetarian	50	71.43%

Menstrual cycle was regular in 78.57% of the cases. In the present study, menstrual history was not taken as significant. This was because some women in the study group were not able to tell history accurately due to general ill health or lack of menstrual cycles with lactational amenorrhea continuing as pregnancy amenorrhea.

In terms of diet, 71.43% of subjects were vegetarian and around 29% were non-vegetarian or with mixed dietary habits. Symptoms suggestive of fatigue was experienced by 64.29% of the anaemic women. The common symptoms were loss of appetite (52.86%), fever (52.86%), dyspnea/palpitation (47.14%), pedal edema (45.71%) and headache/giddiness (40%). In the study subjects, moderate anaemia was 44.28% and severe anaemia was 55.71%. The mean haemoglobin level of all study groups was 7.0 gm%. Maximum haemoglobin value was 8.4 gm% and minimum was 4.6 gm%

Complete blood count with peripheral blood smear in 34.29% was analysed as iron deficiency anaemia and dimorphic anaemia in 61.42%. The current study reported no case of megaloblastic anaemia. G6PD deficiency is not routinely screened, and the current probe encountered no patient with it. Significant bacteriuria was found in 17.14% of study subjects. Anaemia decreases the immunity of the mother and therefore predisposes to infections. Hookworm/roundworm infestations were seen in 5.71%. They were given deworming doses of anthelmintic agents. As shown in Table 2, the three main maternal complications found were sepsis (25.71%), PPH (18.57%), and wound gaping (4.28%).

**Table 2:** Maternal complications in puerperium in the study subjects PPH: postpartum haemorrhage; CCF: congestive cardiac failure

Maternal Outcomes	Number	%
Preterm labour	28	40%
Pre-eclampsia	11	15.71%
Abruptio Placenta	3	4.28%
Placenta Previa	4	5.71%
PPH	13	18.57%
Sepsis	18	25.71%
CCF	4	5.71%
Wound Gaping	3	4.28%

**Figure 1:**
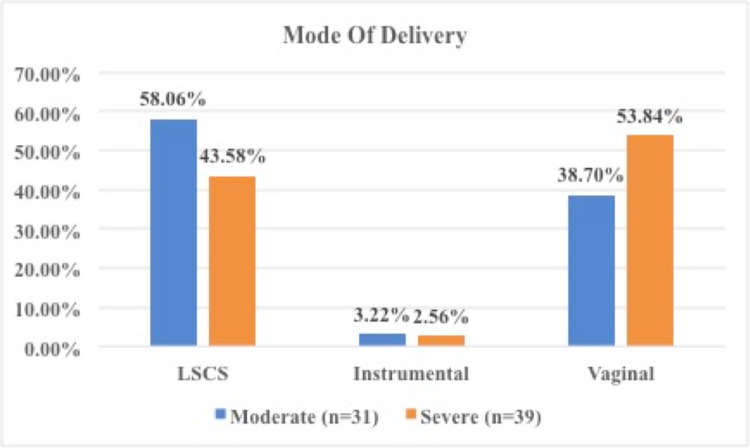
Comparison between severity of anaemia and mode of delivery LSCS: lower segment cesarian section

Of the babies, 35.71% had PTB, 37.14% were LBW, 8.57% had IUD, 15.71% had low APGAR at one minute, 14.28% had low APGAR at five minutes, 7.14% had birth asphyxia, 8.57% had meconium aspiration syndrome (MAS), 17.14% had respiratory distress syndrome (RDS), 47.14% had NICU admission, mostly with severe anaemia (Table 3). As shown in Figure 1, 47.14% were delivered vaginally, 50% were delivered by caesarean section, and 2.86% had instrumental delivery.

**Table 3:** Comparison between fetal outcome and severity of anaemia APGAR: Appearance, Pulse, Grimace, Activity, and Respiration; NICU: neonatal intensive care unit

	Moderate Anemia	Severe Anemia
Intrauterine death	2 (6.45%)	4 (10.25%)
Preterm birth	9 (29.03%)	16 (41.02%)
Low Birth Weight	8 (25.80%)	18 (46.15%)
Low APGAR at one minute	11 (35.48%)	10 (25.64%)
Low APGAR at five minutes	3 (9.67%)	7 (17.94%)
NICU admission	13 (41.93%)	20 (51.28%)
p value >.05		

**Figure 2:**
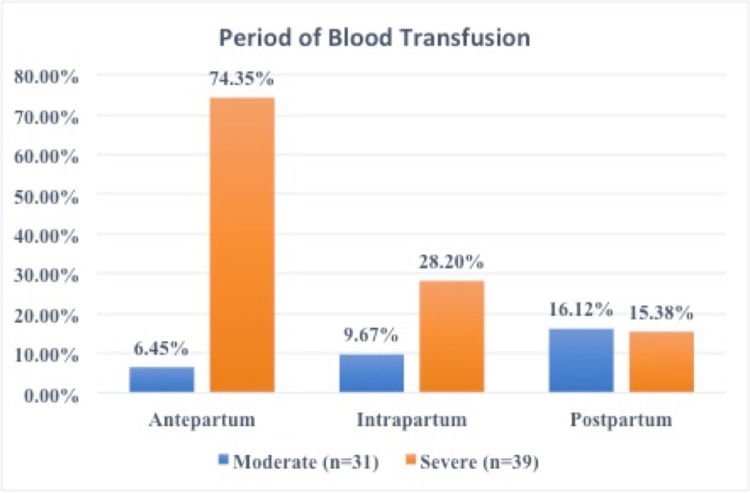
Distribution of cases with varying degrees of anaemia on basis of period of blood transfusion

Of the subjects, 32.85% had IV iron infusion and 70% had a blood transfusion. They were prescribed oral iron afterwards. IV Iron supplementation frequency was lesser as patients were of moderate to severe anaemia and in the last trimester, i.e. near the estimated date of delivery. Time for raising iron to optimal levels before delivery was less in the third trimester and so blood transfusion was the treatment of choice. As shown in Figure 2, antenatal blood transfusion was maximum among severe anaemia, i.e 74.35%. Total postpartum transfusion and intrapartum transfusions were more than the antepartum in moderate anaemia. In severe anaemia, labour complications were already anticipated, hence antepartum transfusions were more than intrapartum and postpartum transfusions.

Maternal morbidity was more with severe anaemia (71.79%). No maternal mortality was reported in the study. Fetal morbidity following the same pattern was more with severe anaemia (53.84%).

## Discussion

Anaemia in pregnancy is an important preventable cause of feto-maternal morbidity and mortality. Studies have demonstrated differences in outcomes in iron deficiency as compared to physiological anaemia of pregnancy. In India, especially in low socioeconomic settings, it is common to see patients with anaemia presenting late in pregnancy with no prior antenatal care. The same was evident in this study, where a vast majority of women were anaemic at delivery. As it is estimated by WHO, there are over 6.3 million perinatal deaths per year globally, almost all of which occur in developing countries, and 27% of them in the least developed countries alone [3].

In the present study, the majority of the study subjects belonged to the age group of 30-34 years (37.14%). Omote et al. observed maximum participants in the 31- 40 years category [4]. The proportion of the pregnant women suffering from anaemia was striking: 9.42% in the age group 30 years or above and maximum in the 20-24 age group (54.54%). The difference is attributable to the fact that all the study subjects in the current observation were multigravida and had more birth spacing.

In our study, 64.28% of the women belonged to the lower socioeconomic status group. In the study by Devi et al., 88.82% of study subjects were of lower socioeconomic class [5]. Although rural and urban populations were approximately of equal frequency in our study, 67% of the total subjects were found to be illiterate. Pundkar et al. found illiteracy to be significant with the development of anaemia (p-value <0.05) [6]. Alternatively, we can say that lower socioeconomic status spells illiteracy. Hence, lack of education is the prevailing reason for poor maternal haemoglobin values. In other words, the prevalence and severity of anaemia decreases as the education level of females increases.

The present study found the commonest type of anaemia to be microcytic hypochromic anaemia mainly followed by dimorphic anaemia. Similar results were found with Singh et al. [7]. A predominantly vegetarian diet might have played a role.

In the current study, 50% of women had a caesarean section and 47.14% had a vaginal delivery. Indications of caesarean section were obstetric only: cephalo-pelvic disproportion, fetal distress, malpresentation, mal-rotation, and non-progress of labour. Strangely, among all severe anaemic women, 53.84% were delivered vaginally. This confirms that the mode of delivery in anaemia is dependent only on obstetric indications.

In the present study, 70% (49/70) subjects were given blood transfusion (46 of these 49 blood transfusions were given to mothers having severe anemia) and 32.85% were given IV iron. The majority (44.28% (31/70)) had blood transfusion due to moderate and severe anaemia in the antepartum period. It is important to highlight here that our institution is designated to aid in blood transfusions to pregnant women as per state government maternal health schemes. Also, a specific group of multiparous women in the third trimester was investigated. At the same time, despite belonging to lower socioeconomic status with high illiteracy, the current research had a higher antenatal booking. Moderate to severe anaemia in close proximity to delivery interval mandates transfusing blood for optimum feto-maternal outcomes. Similarly, a study by Catherine Smith et al. observed more transfusions in moderate to severe anaemia [8].

Preterm labour was experienced by 40% of women. It is comparable from the data of Devi et al. in which 44.68% had preterm labour [9].

Talking about the maternal outcomes, 15.71% had pre-eclampsia, which was comparable with Singh et al. (16.1%) [9]; 4.28% had abruptio placenta, which varied from the data of Devi NB et al. (8.5%) [8]; 18.57% subjects had PPH. A total of 25.80% and 43.58% of women required intrapartum or postpartum blood transfusion in moderate (n=31) and severe (n=39) anaemia, respectively, which was similar to Miglani et al [10]. In the same study, 17% was seen to have sepsis and 5% had CCF [10], while in the current study, 25.71% had sepsis and 5.71% had CCF. In significant contrast, Singh et al. reported heart failure in a meagre 1.4% cases of severe anaemia [7]. Wound gaping was 4.28% in our study, which was comparable with Devi et al. [5]. This surgical site infection (SSI) was treated conservatively with antibiotics, local hygiene, correction of iron deficiency, and good nutrition. All SSIs were subsequently cured. No resuturing was required.

As there is reduced oxygen-carrying capacity due to low haemoglobin, fetal distress ensues. Due to the activation of hypothalamo-pituitary axis of the foetus, the onset of labour occurs, mostly before term. It was observed that 35.71% of the deliveries were PTB and consequently LBW (37.14%). Figueiredo et al. found similar results with a 38% higher risk of having children with low/insufficient weight at birth in pregnant women with anaemia [11]. Our observations of 47.14% NICU admissions were similar to Miglani et al. (35.86%) [11]. The reason for high NICU admission might be that being a tertiary care centre, high risk and complicated deliveries are encouraged to deliver here. Of the babies, 15.71% had a low APGAR score at birth while 8.57% had IUD, which was consistent with Singh et al. (10.9%) [6]. LBW and PTB are interconnected. Preterm babies are more likely to be born LBW and be admitted to the NICU.

Foetus, as well as neonates of anaemic mothers, suffer from a myriad of conditions due to low haemoglobin/low oxygen intrauterine habitat. Distress in utero was evident by birth asphyxia (7.14%), MAS (8.57%), RDS (17.14%), which was comparable with the study of Miglani et al. in which 8.6% had birth asphyxia, 7.5% had MAS, and 13.9% had RDS [10]. Suryanarayana et al. found birth asphyxia in 0.5% of cases [12]. The wide disparity between our observations is because this is a hospital-based study and they did a community-based study. Preterm babies have more risk of RDS, MAS, birth asphyxia, and consequently require more intensive neonatal care.

The limitations of the current analysis are that it is a single hospital-based study. Also, the place of study is a tertiary care setting which usually gets complicated pregnancies, which might be a bias. Future recommendations are to conduct a community-based survey.

## Conclusions

Secondary prevention in multiparous antenatal women can be made possible by early diagnosis and treatment of anaemia. Efforts must be made to diagnose and treat anaemia in the third trimester to decrease length of hospital stay and reduce maternal and neonatal morbidity and mortality. Strategies such as food fortification, mandatory iron, and folic acid supplementation, deworming, and diligent applications of national health programmes should be done. Counseling women, spouses, families, and society on the causes and outcomes of anaemia during pregnancy and after childbirth may help the cause in the long run and enhance public health.
